# Assessment of histologic prognostic factors of resectable rectal cancer: comparison of diagnostic performance using various apparent diffusion coefficient parameters

**DOI:** 10.1038/s41598-020-68328-0

**Published:** 2020-07-14

**Authors:** Hang Li, Guang-wen Chen, Yi-Sha Liu, Hong Pu, Long-lin Yin, Neng-yi Hou, Xiao-li Chen

**Affiliations:** 1 Department of Radiology, Sichuan Academy of Medical Sciences and Sichuan Provincial People’s Hospital, 32# Second Section of First Ring Road, Qingyang District, Chengdu, 610072 Sichuan China; 2 Department of Pathology, Sichuan Academy of Medical Sciences and Sichuan Provincial People’s Hospital, 32# Second Section of First Ring Road, Qingyang District, Chengdu, 610072 Sichuan China; 3 Department of Gastrointestinal Surgery, Sichuan Academy of Medical Sciences and Sichuan Provincial People’s Hospital, 32# Second Section of First Ring Road, Qingyang District, Chengdu, 610072 Sichuan China; 4 Department of Radiology, Affiliated Cancer Hospital of Medical School, University of Electronic Science and Technology of China, Sichuan Cancer Hospital, Chengdu, 610041 China

**Keywords:** Cancer, Gastrointestinal cancer, Colorectal cancer, Rectal cancer

## Abstract

This study is to investigate optimum apparent diffusion coefficient (ADC) parameter for predicting lymphovascular invasion (LVI), lymph node metastasis (LNM) and histology type in resectable rectal cancer. 58 consecutive patients with resectable rectal cancer were retrospectively identified. The minimum, maximum, average ADC and ADC difference value were obtained on ADC maps. Maximum ADC and ADC difference value increased with the appearance of LVI (*r* = 0.501 and 0.495, *P* < 0.001, respectively) and development of N category (*r* = 0.615 and 0.695, *P* < 0.001, respectively). ADC difference value tended to rise with lower tumor differentiation (*r* = − 0.269, *P* = 0.041). ADC difference value was an independent risk factor for predicting LVI (odds ratio = 1.323; *P* = 0.005) and LNM (odds ratio = 1.526; *P* = 0.005). Maximum ADC and ADC difference value could distinguish N0 from N1 category, N0 from N1–N2, N0–N1 from N2 (all *P* < 0.001). Only ADC difference value could distinguish histology type (*P* = 0.041). ADC difference value had higher area under the receiver operating characteristic curve than maximum ADC in identifying LVI (0.828 vs 0.797), N0 from N1 category (0.947 vs 0.847), N0 from N1–N2 (0.935 vs 0.874), and N0–N1 from N2 (0.814 vs 0.770). ADC difference value may be superior to the other ADC value parameters to predict LVI, N category and histology type of resectable rectal cancer.

## Introduction

The determination of prognosis in patients with rectal cancer depends on several factors, such as tumor invasion into and beyond the bowel wall, involvement of the mesorectal fascia (MRF), number of lymph node metastasis (LNM), lymphovascular invasion (LVI) and histology type^1–4^. The current trends in the management of rectal cancer depend on detailed information on the patient’s individual tumor profile. According to National Comprehensive Cancer Network (NCCN) Clinical Practice Guideline, patients with cT1N0M0 stage disease can be treated by transanal endoscopic microsurgery^5^. Radical total mesorectal excision (TME) surgery should be recommended for T1N+M0 stage disease because of high-risk recurrence. Moreover, short-course preoperative radiotherapy can be recommended for patients with rectal cancer (cT1–T3, cN1–N2). If T3N+ patients with negative circumferential resection margin and lower tumor want to receive local excision rather than abdominoperineal resection procedure, they should go for preoperative neoadjuvant therapy. Moreover, LVI, which is defined as cancer cells in peritumoral lymphatic vessels or small nonmuscularized blood vessels or both, has been recognized as an important prognostic determinant in colorectal cancer independent of stage^6^. Higher tumor grade suggests poor prognosis and often require preoperative chemoradiotherapy^7^. At present, the degree of tumor invasion into and beyond the bowel wall and involvement of MRF can be assessed on preoperative MRI or EUS. However, preoperative lymph node staging by morphologic features showed poor performance with a wide accuracy range of 45–85%^8–10^. LVI and histology type are traditionally assessed by postoperative histologic evaluation.

The apparent diffusion coefficient (ADC) is used to quantify water diffusion, and some of rectal cancers show low ADC values compared with normal tissue. However, the methods for measuring ADC differ among reported studies. Some studies indicated that minimum ADCs may be an optimal DWI single parameter for differentiation between breast malignant and benign lesions because minimum ADC values corresponding to the sites of highest cellularity within heterogeneous tumors^11^. Murakami et al. reported that combination of minimum ADCs and ADC difference values facilitated the accurate grading of astrocytic tumor^12^. Other studies suggested that DWI of malignant tumor reflects not only cell density but also architectural variations of the stroma^13^. Therefore, we hypothesized that these ADC parameters may help predict LVI, LNM and histology type in resectable rectal cancer. This study aimed to investigate the utility of minimum ADC, maximum ADC, ADC difference value and average ADC to find optimum ADC parameter for predicting LVI, LNM and histology type.

## Materials and methods

This retrospective study was approved by the Institutional Review Board of Sichuan Provincial People’s Hospital, and written informed consent was waived. All methods were performed in accordance with the relevant guidelines and regulations.

120 consecutive patients with rectal cancer confirmed by endoscopic biopsy underwent preoperative MRI from August 2018 to April 2019. The exclusion criteria: preoperative neoadjuvant chemoradiotherapy (n = 40); tumors is not found on MRI (n = 5) and extremely small tumors (< 1 cm^3^; n = 4); poorly image quality (n = 5); postoperative pathologic neuroendocrine tumor (n = 4); mucinous adenocarcinoma which exhibit high ADC values because of a very low cellular density (n = 4). Finally, fifty-eight patients (male: 36, female: 22, female/male ratio: 0.61, mean age, 61.58 ± 12.97 years old; range, 24–85 years old) were enrolled in this study. There were 7 patients with T1N0M0, 12 patients with T2N0M0, 6 patients with T2N1M0, 1 patient with T2N2M0, 11 patients with T3N0M0, 6 patients with T3N1M0 and 15 patients with T3N2M0.The interval between surgery and MRI examination was in one week.

### Imaging protocol

20 mg of scopolamine butylbromide was intramuscular injected to each patient (Buscopan, Boehringer Ingelheim) approximately 30 min prior to MRI to inhibit bowel motion. For correctly determining T category, patients had never underwent rectal distention before MRI scan. Pelvic scan was performed. Sagittal and coronal T2-weighted images using a 1.5-T MR scanner (Magnetom Avanto, Siemens Healthcare) were performed: TR/TE, 3,500/87; FOV, 25 mm^2^; matrix size, 320 × 240; section thickness, 3 mm; and intersection gap, 0.6 mm. We did not use fat saturation in this study. Axial (perpendicular to the tumor axis) T2-weighted TSE sequence were performed using following parameters: TR/TE, 4,233/87; FOV, 25 mm^2^; matrix size, 320 × 240; section thickness, 4 mm; and intersection gap, 0.6 mm. The axial DWI was angulated perpendicular to the tumor axis using a single-shot DWI sequence. The DWI parameters were as follows: TR/TE, 5,600/78; FOV, 360 mm^2^; matrix size, 115 × 192; section thickness, 5 mm; and b = 0 and 800 s/mm^2^. ADC maps of isotropic images were created automatically by the device.

### Image analysis

**Figure 1:**
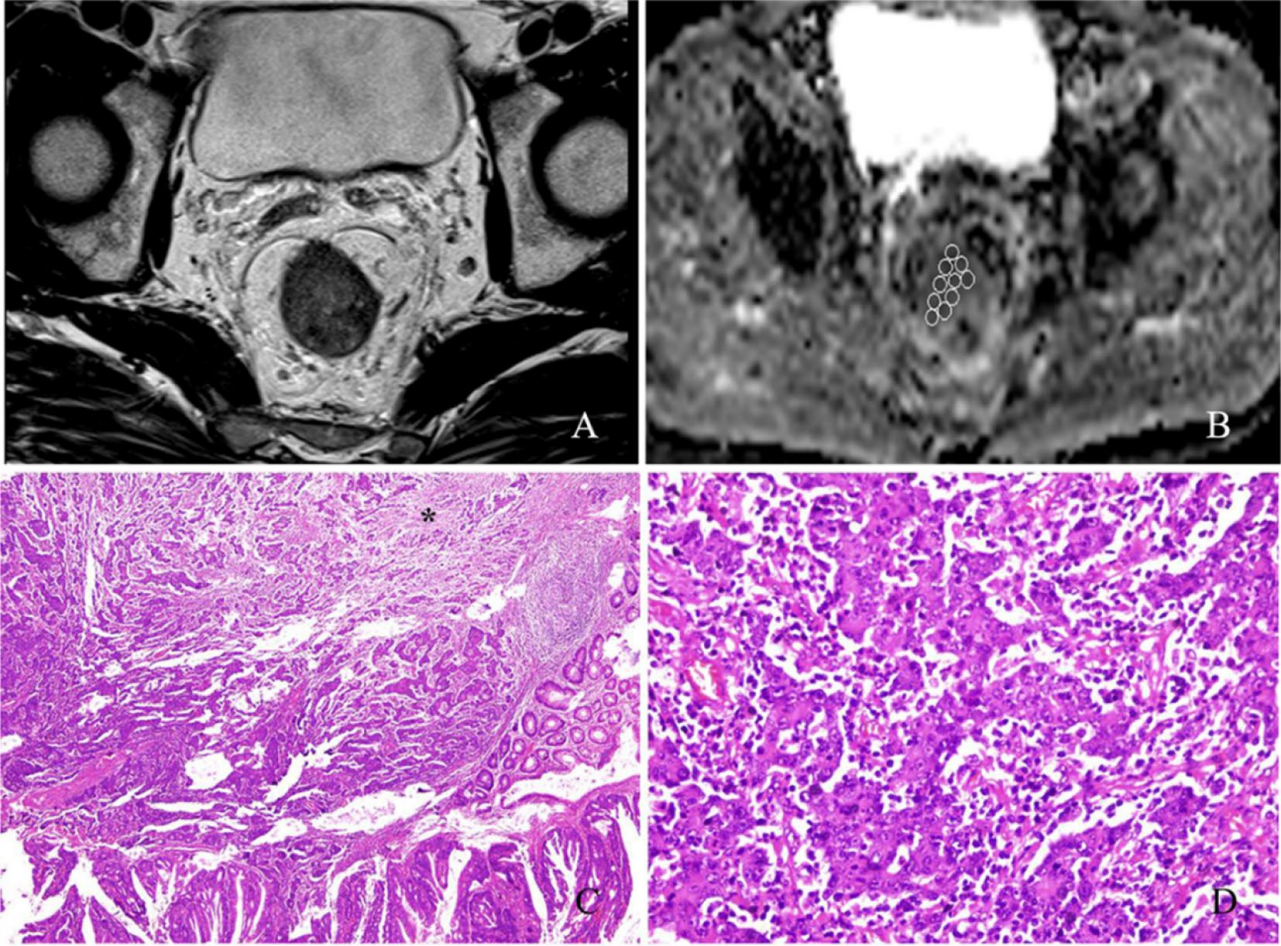
A 60-year-old woman with moderately differentiated rectal cancer. (**A**) T2-weighted image shows a clear delineation of the tumor. (**B**) Multiple regions of interest (ROIs) of 13–20 mm^2^ were placed within the mass lesion on ADC map, and ADC values were obtained as follows: minimum ADC, 0.368 × 10^−3 ^mm^2^/s; maximum ADC, 1.235 × 10^−3 ^mm^2^/s; ADC difference value, 0.867 × 10^−3 ^mm^2^/s; average ADC, 0.654 × 10^−3 ^mm^2^/s. (**C**) Photomicrograph of histologic specimen shows intratumoral heterogeneity in which high- and low cell density areas coexist and marked interstitial fibrosis (single asterisk) (original magnification × 40; hematoxylin–eosin [H–E] stain). (**D**) Photomicrograph of histologic specimen shows many lymphocytes, plasmacytes, and neutrophils in the interstitial space of cancer glands (original magnification × 400; HE stain).

Two radiologists (6 and 7 years of experience in reading rectal MRI) who were blinded to the pathological results independently delineated the regions of interest (ROIs) manually on all consecutive tumor slices of the ADC map (b = 800 s/mm^2^). According to the previous study^11^, initially, multiple ROIs with 13–40 mm^2^ were drawn as many as possible within the tumor referring to T2-weighted images for determining tumor boundaries (Fig. 1). All ROIs were carefully drawn inside the tumor in order to avoid partial volume effects. T2-weighted images were used to avoid obvious cystic and necrotic components. Subsequently, the average ADC (× 10^−3^ mm^2^/s) for each tumor slice was calculated by taking the mean of the summed mean values of all ROIs on each tumor slice of the DW images. And then these calculated average ADC values from all consecutive tumor slices were averaged as the whole tumor average ADC value for statistical analysis. The present definition of minimum ADC is the average ADC in the area with the minimal average. The ROI with the minimum ADC value (× 10^−3^ mm^2^/s) was selected from the multiple ROIs within the targeted mass on each tumor slice. That ADC value was regarded as the minimum ADC for each tumor slice. And then these selected minimum ADC values from all consecutive tumor slices were averaged as the whole tumor minimum ADC value for statistical analysis. In the same way, the ROI with the maximum ADC value (× 10^−3^ mm^2^/s) was selected from the multiple ROIs within the targeted mass on each tumor slice. That ADC value was regarded as the maximum ADC for each tumor slice. And then these selected maximum ADC values from all consecutive tumor slices were averaged as the whole tumor maximum ADC value for statistical analysis. To reflect tumor heterogeneity, ADC difference value (× 10^−3^ mm^2^/s) was defined as the difference between maximum and minimum ADC value on each tumor slices. The selected ADC difference values from all consecutive tumor slices were averaged as the whole tumor ADC difference value for statistical analysis.

To verify the interobserver reproducibility of ADC measurements, when the percentage coefficient of variation (CV) for the two radiologists’ measurements exceeded 10%, one more measurement was performed by the same two radiologists and the mean of the four measurements was regarded as the final ADC parameters^14^. If the percentage CV for the two radiologists’ measurements was less than 10%, these differences were considered negligible, and only the first radiologist measurements were used as the final ADC parameters.

For qualitative assessment, LNM was evaluated on T2-weighted images by two radiologists working in consensus. LNM on the T2-weighted images were predefined as LNM with short axis diameters greater than 8 mm or indistinct, spiculated borders with mottled heterogeneous signal intensity regardless of the short axis diameter^15^. Pathological N category served as the reference standards, which were generated according to the TNM staging system.

### Surgical histologic findings

58 patients underwent TME and at least 12 regional lymph nodes should be examined. The fresh specimens were sent to the pathology laboratory. One pathologist with 7 years of experience performed the pathologic evaluation. Each specimen was immersed in buffered formalin saline for at least 72 h. The TME specimen was sectioned transversely stepwise at 3-mm intervals. The slices were laid out and photographed. The depth of tumor invasion, histology type, MRF invasion and presence of LNM and LVI were reported.

### Statistical analysis

Statistical analyses were performed using the Statistical Package for the Social Sciences (version 17.0, SPSS). A *P* value < 0.05 was considered significant. The relationships between ADC parameters and LVI, LNM and histology type were tested by Spearman rank correlation analyses. The correlation between clinicopathologic factors and LNM and LVI, and between ADC parameters and LNM and LVI were evaluated using a chi-square test (or Fisher exact test when appropriate). Multivariate logistic regression analyses were performed to find the risk factors for LNM and LVI. Nonparametric Mann–Whitney tests were used to compare the ADC parameters between N categories. If significant positive results were present, receiver operating characteristic (ROC) curve analysis was performed to identify the cutoff ADC parameters for identifying LVI, N category and histology type.

## Results

### Interobserver variability of ADC measurements

In assessments of interobserver variability for ADC measurements, the mean CV was 12% (range, 1–25.4%) for minimum ADC value, 6.5% (range, 0.3–13.7%) for average ADC value, 16% (range, 0.8–30.0%) for maximum ADC value, and 7.6% (range, 0.8–18.6%) for the ADC difference value. The CV exceeded 10% in minimum ADC value measurements for 20 patients, average ADC value measurements for 6 patients, maximum ADC value measurements for 26 patients, and ADC difference value measurements for 7 patients. Therefore, for these patients, one more measurement was performed by the same radiologists, and the mean of the four measurements was regarded as the final ADC parameters.

### Univariate and multivariate analysis

The results of univariate analysis correlating clinicopathologic factors and ADC parameters with LNM and LVI are shown in Table 1 and Table 2. LVI was present more frequently in patients with LNM than without LNM involvement (*P* < 0.001), with maximum ADC ≥ 1.279 than < 1.279 (*P* = 0.002) and in those with ADC difference value ≥ 0.656 than < 0.656 (*P* < 0.001). LNM was present more frequently in patients with deeper tumors than in those with low tumor depth (*P* = 0.006), with well or moderately differentiated than poorly differentiated (*P* = 0.005), with LVI than without LVI (*P* < 0.001), with maximum ADC ≥ 1.279 than < 1.279 (*P* = 0.001) and in those with ADC difference value ≥ 0.656 than < 0.656 (*P* < 0.001).

**Table 1:** Univariate analysis of clinicopathologic factors and apparent diffusion coefficient (ADC) values correlated with lymphovascular invasion in resectable rectal cancer.

Variable	Lymphovascular invasion		*P*
	Present (n = 13)	Absent (n = 45)	
Median age ± SD	62.07 ± 13.12	62.02 ± 13.04	0.638
**Sex**			0.965
Male	8 (61.5)	28 (62.2)	
Female	5 (38.5)	17 (37.8)	
**Anatomic distribution**			0.947
Upper	6 (46.1)	23 (51.1)	
Middle	4 (30.8)	13 (28.9)	
Lower	3 (23.1)	9 (20.0)	
**Histology type**			0.67
Well or moderate	10 (76.9)	37 (79.1)	
Poor	3 (23.1)	8 (20.9)	
**T category**			0.061
T1	0 (0)	7 (15.5)	
T2	2 (15.4)	17 (37.8)	
T3	11 (84.6)	21 (46.7)	
**Minimum ADC (× 10**^**−3**^** mm**^**2**^**/s)**			0.529
< 0.616	8 (61.5)	21 (46.7)	
≥ 0.616	5 (38.5)	24 (53.3)	
**Maximum ADC (× 10**^**−3**^** mm**^**2**^**/s)**			0.002
< 1.279	1 (7.7)	28 (62.2)	
≥ 1.279	12 (92.3)	17 (37.8)	
**ADC difference value (× 10**^**−3 **^**mm**^**2**^**/s)**			< 0.001
< 0.656	0 (0)	29 (64.4)	
≥ 0.656	13 (100)	16 (35.6)	
**Average ADC (× 10**^**−3**^** mm**^**2**^**/s)**			0.753
< 0.906	7 (53.8)	22 (48.9)	
≥ 0.906	6 (46.2)	23 (51.1)	
**LNM**			< 0.001
Positive	13 (100)	17 (37.8)	
Negative	0 (0)	28 (62.2)	

Values are number of patients with percentages in parentheses.

*LNM* lymph node metastasis.

**Table 2:** Univariate analysis of clinicopathologic factors and apparent diffusion coefficient (ADC) values correlated with lymph node metastasis in resectable rectal cancer.

Variable	Lymph node metastasis		*P*
	Present (n = 28)	Absent (n = 30)	
Median age ± SD	59.93 ± 14.47	63.13 ± 11.43	0.352
**Sex**	19 (67.8)	17 (56.7)	0.368
Male	9 (32.2)	13 (43.3)	
Female			
**Anatomic distribution**			0.836
Upper	14 (50.0)	15 (50.0)	
Middle	9 (32.1)	8 (26.7)	
Lower	5 (17.9)	7 (23.3)	
**Histology type**			0.005
Well or moderate	18 (64.2)	29 (96.7)	
Poor	10 (35.8)	1 (3.3)	
**T category**			0.006
T1	0 (0)	7 (23.3)	
T2	8 (28.6)	11 (36.7)	
T3	20 (71.4)	12 (40.0)	
**Minimum ADC (× 10**^**−3**^** mm**^**2**^**/s)**			0.431
< 0.616	12 (42.8)	17 (56.7)	
≥ 0.616	16 (57.2)	13 (43.3)	
**Maximum ADC (× 10**^**−3**^** mm**^**2**^**/s)**			0.001
< 1.279	7 (25.0)	22 (73.3)	
≥ 1.279	21 (75.0)	8 (26.7)	
**ADC difference value (× 10**^**−3**^** mm**^**2**^**/s)**			< 0.001
< 0.656	3 (10.7)	26 (86.7)	
≥ 0.656	25 (89.3)	4 (13.3)	
**Average ADC (× 10**^**−3**^** mm**^**2**^**/s)**			0.189
< 0.906	11 (39.2)	18 (60.0)	
≥ 0.906	17 (60.8)	12 (40.0)	
**Lymphovascular invasion**			< 0.001
Positive	11 (39.3)	2 (6.7)	
Negative	2 (60.7)	43 (93.3)	

Values are number of patients with percentages in parentheses.

*SD* standard deviation.

According to multivariate analysis, ADC difference value was an independent risk factor for predicting LVI (odds ratio = 1.323; 95% CI, 1.156–1.508; *P* = 0.005) and LNM (odds ratio = 1.526; 95% CI, 1.211–1.863; *P* = 0.005).

### Correlation of histology type, lymphovascular invasion and lymph node metastases with ADC parameters

**Figure 2:**
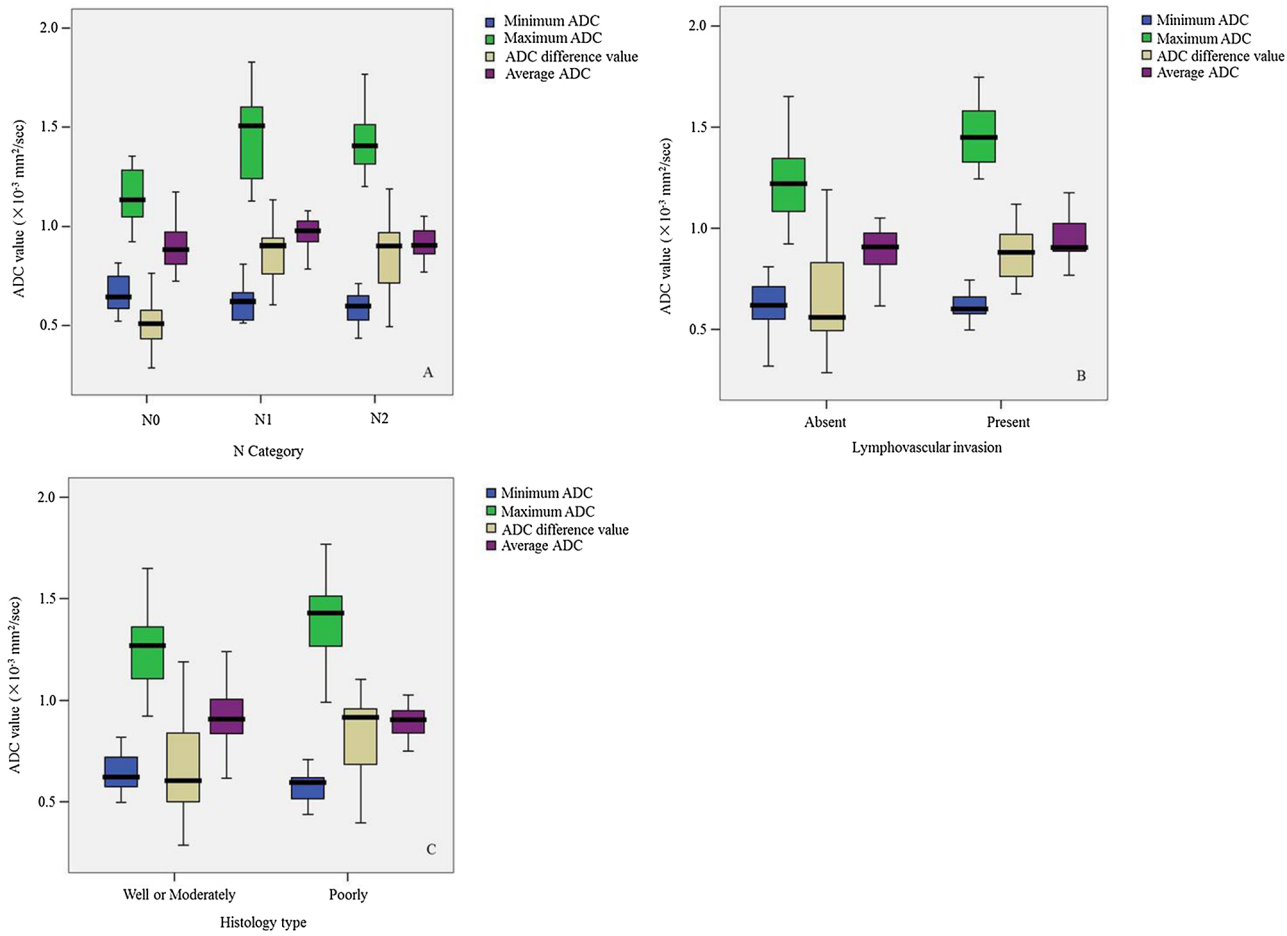
Box plots show association of the minimum ADC, maximum ADC, ADC difference value and average ADC with N category (**A**), lymphovascular invasion (**B**) and histology type (**C**) in resectable rectal cancer.

Maximum ADC and ADC difference value increased with LVI present (*r* = 0.501 and 0.495, *P* < 0.001, respectively) and with the increasing of N category (*r* = 0.615 and 0.695, *P* < 0.001, respectively). ADC difference value tended to rise with lower tumor differentiation (*r* = −0.269, *P* = 0.041). Table 3 and Fig. 2 show the relationship between histologic prognostic factors and ADC parameters. Both maximum ADC and ADC difference value could predict LVI (both *P* < 0.001). Only ADC difference value could distinguish histology type (*P* = 0.041). Minimum ADC could distinguish N0 from N1–N2 category (*P* = 0.026) and average ADC could distinguish N0 from N1 category (*P* = 0.021). Both maximum ADC and ADC difference value could distinguish N0 from N1 category, N0 from N1–N2, N0–N1 from N2 (all *P* < 0.001), but none of ADC parameters could distinguish N1 from N2 category (all *P* > 0.05).

**Table 3:** Apparent diffusion coefficient (ADC) measurement of resectable rectal cancer in patients stratified by N category, lymphovascular invasion and histology type.

Characteristic	ADC measurement (× 10^−3^ mm^2^/s)			
N Category	Minimum ADC	Maximum ADC	ADC difference value	Average ADC
N0	0.645 (0.583, 0.752)	1.135 (1.048, 1.288)	0.510 (0.428, 0.579)	0.883 (0.803, 0.98)
N1	0.621 (0.522, 0.681)	1.507 (1.234, 1.623)	0.903 (0.724, 0.948)	0.977 (0.915, 1.028)
N2	0.599 (0.522, 0.655)	1.407 (1.302, 1.536)	0.902 (0.697, 0.969)	0.905 (0.851, 0.995)
N0–N1	0.628 (0.575, 0.730)	1.219 (1.072, 1.347)	0.555 (0.495, 0.780)	0.908 (0.829, 1.004)
N1–N2	0.603 (0.522, 0.655)	1.448 (1.278, 1.558)	0.903 (0.704, 0.966)	0.934 (0.886, 1.018)
**Lymphovascular invasion**				
Positive	0.603 (0.513, 0.684)	1.471 (1.344, 1.622)	0.868 (0.733, 0.963)	0.905 (0.885, 1.024)
Negative	0.621 (0.552, 0.716)	1.220 (1.079, 1.349)	0.559 (0.495, 0.837)	0.908 (0.821, 0.981)
**Histology type**				
Well or moderate	0.595 (0.513, 0.635)	1.429 (1.258, 1.558)	0.916 (0.680, 0.961)	0.905 (0.834, 0.985)
Poor	0.622 (0.569, 0.721)	1.270 (1.096, 1.370)	0.605 (0.497, 0.845)	0.908 (0.831,1.004)

Data are medians with 25th and 75th percentiles in parentheses, respectively.

### ROC analyses of ADC parameters for identifying histology type, lymphovascular invasion and lymph node metastases

**Figure 3:**
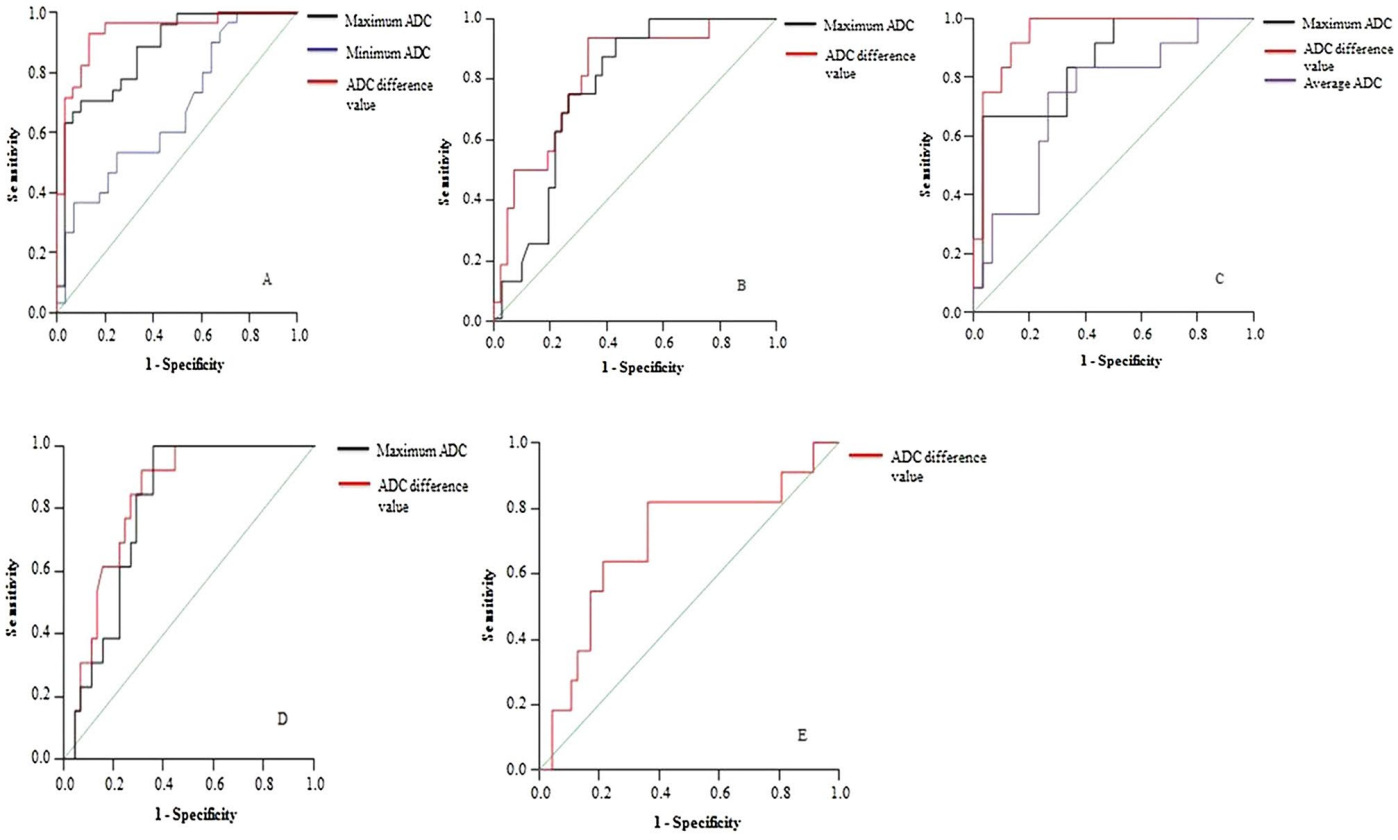
Receiver operating characteristic curves (ROC) curves. Diagonal line is line of reference. (**A**) differentiation of N0 from N1–N2 category with maximum ADC (black line), minimum ADC (blue line) and ADC difference value (red line). (**B**) differentiation of N0–N1 from N2 category with maximum ADC (black line) and ADC difference value (red line). (**C**) differentiation of N0 from N1 category with maximum ADC (black line), ADC difference value (red line) and average ADC (purple line). (**D**) prediction of lymphovascular invasion with maximum ADC (black line) and ADC difference value (red line). (**E**), differentiation of well or moderately differentiated from poorly differentiated with ADC difference value (red line).

As illustrated in Table 4 and Fig. 3, the ROC AUC values were higher for ADC difference value than maximum ADC (0.828 vs 0.797) for predicting LVI. ADC difference value had similar sensitivity with` maximum ADC for LVI (84.6% vs 84.6%). ROC AUC values were higher for ADC difference value than average ADC (0.947 vs 0.731) and maximum ADC (0.947 vs 0.847) for distinguishing N0 from N1 category. The ROC AUC values were higher for ADC difference value than maximum ADC for distinguishing N0 from N1–N2 category (0.935 vs 0.874) and N0–N1 from N2 (0.814 vs 0.770). Only ADC difference value could distinguish well or moderately differentiated from poorly differentiated with AUC of 0.700, sensitivity of 72.7% and specificity of 64.8%.

**Table 4:** Receiver operating characteristic (ROC) analysis of apparent diffusion coefficient (ADC) value of resectable rectal cancer for predicting lymphovascular invasion (LVI), Histology type and N Category.

ADC cutoff (× 10^−3^ mm^2^/s)	Comparison	AUC	Sensitivity (%)	Specificity (%)
**Minimum ADC**				
0.616	N0 versus N1–N2	0.671	60	58.1
**Maximum ADC**				
1.332	LVI ( +) versus (−)	0.797	84.6	71
1.223	N0 versus N1	0.847	83.3	67.7
1.271	N0 versus N1–N2	0.874	78.6	70
1.343	N0–N1 versus N2	0.770	75	73
**ADC difference value**				
0.703	LVI ( +) versus (−)	0.828	84.6	73.3
0.623	N0 versus N1	0.947	91.7	86.7
0.642	N0 versus N1–N2	0.935	92.9	86.7
0.672	N0–N1 versus N2	0.814	81.3	73
0.684	Well or moderate versus poor	0.700	72.7	64.8
**Average ADC**				
0.928	N0 versus N1	0.731	75	74.3

*AUC* area under the ROC curve.

### Qualitative analysis for identifying lymph node metastases

According to pathological N category, there were 30 patients in N0 stage, 12 patients in N1 category and 16 patients in N2 category. When the group with pathological N1–N2 category was used as the reference, the MRI sensitivity was 72.4%, with a specificity rate of 75.8%. When the group with pathological N2 category was used as the reference, the MRI sensitivity was 32%, with a specificity rate of 75%.

## Discussion

In this study, we found that ADC difference value was an optimal parameter for identifying LVI, LNM and histology type. Our preliminary data suggest that ADC difference value was associated with LVI, N category and histology type in rectal cancer. Specifically, ADC difference value could help differentiate patients with LVI from without LVI, N0 from N1, N0 from N1–N2, N0–N1 from N2, and well or moderately differentiated from poorly differentiated.

One important methodologic concern is ROI placement. Most previous study placed the whole tumor ROI or three round/oval-shaped ROIs within tumor^16–18^. In heterogeneous rectal tumors, volume averaging with surrounding tissues should be considered. Because large ROIs obscure measurement of minimum ADC foci and ADC measurements obtained on a pixel-by-pixel basis may be strongly affected by the artifacts of DW imaging and by ADC map misregistration. Two published studies breast mass suggested that ADC value should be selected from multiple small ROIs on ADC map, not the ADC value corresponding to a pixel in one ROI surrounding the mass^11,19^. However, ADC values obtained in these studies were not measured on all slices from the masses, and the ADC value could not reflect the character of the entire mass. In contrast to previous studies, we manually delineated multiple small ROIs on the whole tumor.

It has been suggested that the regions with minimum ADC value corresponding to the foci with highest cell density within heterogeneous tumors, reflecting the invasive nest sites, and, therefore, these sites may be of diagnostic value in identifying high-grade tumor components^20–22^. Based on these previous studies^11,12,19^, we hypothesize that minimum ADC value could also be carried out to identify histologic prognostic factors in rectal cancer. However, our studies confirmed that minimum ADC value could not identify LVI and histology type in rectal cancer. Although it can differentiate N0 from N1–N2 category, the diagnostic efficacy was not very high with AUC of 0.671. The reason may be that minimum ADC value was affected by not only the cancer cell density, but also the entire organized cell density, including the stroma of the rectum. Akashi et al. reported that some patients with well differentiated rectal adenocarcinoma had a lower ADC value when compared with the ADC value of other patients with the same differentiation grade adenocarcinoma^13^.They further found that lower ADC cases had more fibroblasts, lymphocytes, plasmacytes, and neutrophils in the interstitial space of the tumor than other patients^13,23,24^. Our pathological finding was also consistent with these previous studies (Fig. 1D). Moreover, other studies indicated that tissue fibrosis was associated with lower ADC values in patients with liver or esophageal cancer^23,25^. Therefore, in addition to cell density, interstitial inflammatory infiltration and tissue fibrosis may also reduce the ADC value in the rectal cancer.

Many studies have focused on the correlation between average ADC and prognostic factors in rectal cancer. Sun et al. reported that average ADC is lower with less differentiated rectal tumors but with no statistically significant difference^26^. They also indicated that the average ADC could not differentiate N category. Another study showed there was no correlation between the average ADC and LVI^16^. Our studies also showed that the average ADC could not predict LVI and poorly differentiated in rectal cancer. Although average ADC can differentiate N0 from N1 category, it can’t differentiate the other N category. The probable reason could be that the average ADC could not reveal the state of cancer interstitial tissue containing inflammatory cells, including tumor-infiltrating lymphocytes^13^. However, Curvo-Semedo et al. found that the average ADC could differentiate histology type and N category in rectal cancer^16^. This may be because N category and histology type used in this study was different from our study. In that study, N category was divided into cN0 and cN+, and histology type was divided into five grades. A large ROI including as much of the solid tumor area as possible was selected in the study, which will affect ADC measurements by the artifacts of DW imaging and by ADC map misregistration.

Previous study had investigated that maximum ADC value of uterine cervical cancer may represent the grade of tumor differentiation and provide valuable information on tumor microcirculation and perfusion, thus allowing a promising new method of non-invasively assessing the pathological grade^27^. Horvat et al. also reported that maximum ADC value could distinguish estrogen receptor-positive and progesterone receptor-positive breast cancer status with greater AUCs^28^. In the present study, we found that maximum ADC value could help differentiate patients with LVI from without LVI, N0 from N1, N0 from N1–N2, N0–N1 from N2 rectal cancer. In theory, maximum ADC value that has decreased cellularity is expected to have negative correlation with LVI and LNM. However, our study indicated that maximum ADC value increased with LVI present and with the increasing of N category. The pathological basis of these findings could be that vessels are known to have increased permeability in malignant tumors because of the loss of membrane integrity^29,30^. Free displacement of blood from vessel to tumor can happen easily, and, therefore, increase the proportion of total extracellular fluid. In the present study, high vascularity in tumor with the increasing amount of extracellular fluid seemed to overcome restricted diffusion related to high cellularity. Therefore, we can conclude that neovascularity in rectal cancer may impact more the ADC values than cellularity, which explains maximum ADC value having positive correlation with LVI and LNM.

The present definition of ADC difference value is the difference between maximum and minimum ADC value. To our knowledge, there have been few studies of ADC difference value for predicting histologic prognostic factors. Previous studies reported that ADC difference value could help predict malignant breast masses, and could distinguish grade 2 and grade 3 astrocytic tumors^11,12^. In this study, we found that ADC difference value was a potentially more promising imaging indicator for identifying LVI, N0 from N1, N0 from N1–N2, and N0–N1 from N2 category. Only ADC difference value could distinguish histology type. The possible explanation could be that ADC difference value reflects the degree of pathologic heterogeneity of tumor, and it takes both the minimum ADC and maximum ADC into consideration. We conclude that the larger the ADC difference value, the more obvious pathologic heterogeneity of tumor appearing, and the more frequent the incidence of LNM, LVI and poorly differentiated.

Compared with the accuracy of MRI in T staging, the accuracy of MRI in assessing LNM in rectal cancer is less accurate. As a large proportion of metastatic lymph nodes in rectal cancer measure less than 5 mm, size is not a reliable criterion^31^. Two meta-analyses reported suboptimal sensitivities and specificities in the range of 55–78% for LNM with standard T2-weighted images^32,33^. Our studies also confirmed that combined node size with morphological characteristic on MRI could not improve the diagnostic efficacy for identifying LNM. It was interesting that the sensitivity was decreased to 32% when we carried out those qualitative parameters for identifying N2 category. In this study, we found that ADC difference value could predict N1–N2 category with sensitivity of 92.9% and specificity of 86.7%, and predict N2 category with moderate sensitivity of 81.3% and specificity of 73%.

There were some limitations. First, the size and shape of ROIs were restricted by the tumor: the ROIs were ranged from 13 to 40 mm^2^ (mean, 24 mm^2^) with a minimum size of 13 mm^2^, which was approximately equivalent to eight pixels. Further studies are needed to verify the correlation between ADC values and ROIs size and to find the optimal size ROI for measurements of ADC values of rectal cancer. Second, although we utilized T2-weighted images to prevent contamination of cystic and/or necrotic components, minor contamination might not be avoided owing to the relatively large slice thickness of T2-weighted images, resulting in suboptimal accuracy of the measurement. Third, the ADC is calculated from only two b-values. Therefore, multiple b-values should be performed in the future study. In addition, because there were only 3 patients with well differentiated, we combined well differentiated and moderately differentiated. Therefore, a much larger sample size is needed to evaluate histology type in rectal cancer.

In conclusion, our results suggest that maximum ADC and ADC difference value are associated with LVI and LNM, and only ADC difference value was correlated with histology type. Employing ADC difference value showed higher diagnostic accuracy in the prediction of histologic prognostic factors in resectable rectal cancer. Consequently, ADC difference value may help identify patients potentially eligible for neoadjuvant treatment or operation.

## Data Availability

Data sharing is not applicable to this article.
